# Haemophagocytic Lymphohistiocytosis Triggered by Visceral Leishmaniasis Masquerading as Epstein-Barr Virus (EBV)-Associated Haemophagocytic Lymphohistiocytosis in Rheumatoid Arthritis: A Case Report and Literature Review

**DOI:** 10.7759/cureus.116027

**Published:** 2026-09-09

**Authors:** Frederik Fleurinck, Liesbet Henckaerts, Mariëlle Beckers, Eef Vanerwegen, Patrick Verschueren

**Affiliations:** 1 Department of Rheumatology, University Hospitals Leuven, Leuven, BEL; 2 Department of General Internal Medicine, University Hospitals Leuven, Leuven, BEL; 3 Department of Microbiology, Immunology and Transplantation, KU Leuven, Leuven, BEL; 4 Department of Hematology, University Hospitals Leuven, Leuven, BEL; 5 Department of Oncology, Laboratory of Experimental Hematology, KU Leuven, Leuven, BEL

**Keywords:** epstein–barr virus (ebv), haemophagocytic lymphohistiocytosis (hlh), rheumatoid arthritis, systemic immunosuppression, visceral leishmaniasis (vl)

## Abstract

Haemophagocytic lymphohistiocytosis (HLH) is a life-threatening hyperinflammatory syndrome caused by uncontrolled immune activation. In adults, it is usually secondary to an underlying condition, such as infection, malignancy, or autoimmune disease. Identifying this trigger is essential, as treatment depends on the cause. Visceral leishmaniasis (VL) is a systemic parasitic infection that may mimic or trigger HLH, particularly in immunosuppressed patients. We report a 59-year-old man with seropositive rheumatoid arthritis, for which he received immunosuppressive treatment with methotrexate and etanercept. For eosinophilic asthma, he was treated with benralizumab. Four months after travelling to the Valencia region in Spain, the patient presented with high fever, marked splenomegaly and pancytopenia. Further laboratory findings showed progressive hyperferritinaemia, hypofibrinogenaemia, and hypertriglyceridaemia, together with haemophagocytosis on bone marrow examination, which supported the diagnosis of secondary HLH. Because Epstein-Barr virus (EBV) DNA was detected in peripheral blood, EBV-associated HLH was initially suspected, and treatment was started with rituximab, targeting potentially EBV-infected B cells, and dexamethasone to suppress the hyperinflammatory response. Further infectious work-up, however, revealed a strongly positive *Leishmania* serology. *Leishmania* polymerase chain reaction on the original bone marrow sample subsequently confirmed VL, despite the absence of amastigotes on Giemsa-stained microscopy. Treatment was redirected to liposomal amphotericin B; further HLH-directed immunosuppressive therapy was withheld, and corticosteroids were tapered. The patient became afebrile and showed progressive recovery of cytopenias and inflammatory markers. Detectable EBV DNA should not preclude investigation for alternative infectious triggers of HLH. In immunosuppressed patients with compatible epidemiological exposure, VL should remain in the differential diagnosis even when bone marrow microscopy is negative, as serology and molecular testing may be required for diagnosis.

## Introduction

Haemophagocytic lymphohistiocytosis (HLH) is a rare but life-threatening hyperinflammatory syndrome, caused by uncontrolled immune activation. It may be primary (pHLH) due to genetic defects. In adults, however, it is usually secondary (sHLH), being triggered by infections (iHLH: viruses, bacteria, and parasites), malignancy (mHLH: lymphoproliferative diseases), or autoimmune diseases [1]. Infection is found to be the main trigger for HLH in patients on biologic therapy [2-4], as in patients not receiving immunosuppressive drugs [1], because the clinical presentation of HLH overlaps with sepsis, malignancy, and severe autoimmune diseases; recognition of HLH and identification of the underlying cause may be delayed, with potentially serious consequences [1].

Leishmaniasis is a vector-borne infection caused by intracellular protozoa of the genus *Leishmania* and transmitted by infected female phlebotomine sandflies [5-7]. Visceral leishmaniasis (VL), the systemic form of the disease, typically presents with prolonged fever, constitutional symptoms, hepatosplenomegaly, and cytopenias [5-7]. In the Mediterranean region, *Leishmania infantum* is the predominant causative species [6]. VL can also trigger secondary HLH, and the two conditions share several clinical and laboratory features [6,8]. VL is fatal in more than 95% of cases if left untreated [5-7].

In Belgium, a non-endemic area, leishmaniasis remains uncommon [9]. In 2024, the national reference laboratory reported 39 cases of leishmaniasis, the highest annual number ever recorded; all were imported, with 17 cases acquired in the Mediterranean region, including 15 in Spain, and *L. infantum* was the most frequently identified species [9]. There is no specific data available on the incidence of VL-HLH in Belgium. A retrospective, monocentric study by Yildiz et al. reported no cases of VL-HLH over a 10-year period at a tertiary care centre in Brussels, Belgium [10].

Immunosuppression is an important risk factor for clinically overt VL [7,11]. Numerous cases have repeatedly been reported during tumour necrosis factor-alpha (TNF-α) inhibition [5,11-21]. Reactivation after starting anti-TNF therapy [5,13] or after remote exposure (the incubation period can range from weeks to several years) [6,7,22] has also been described. In some cases, the exposure remains unclear [13,22]; for example, in an old Belgian case of leishmaniasis without a prior travel history, blood transfusion has been implicated [23].

We present a case of HLH triggered by VL in a patient with rheumatoid arthritis (RA) on anti-TNF therapy for two years, four months after travelling to the Valencia region of Spain. The diagnostic process was complicated by detectable Epstein-Barr virus (EBV) DNA, initially suggesting possible EBV-associated HLH, and by negative bone marrow microscopy for *Leishmania*, with the diagnosis ultimately established by serology and polymerase chain reaction (PCR).

## Case presentation

We report the case of a 59-year-old male patient on methotrexate 20 mg weekly and etanercept 50 mg weekly since June 2023 for seropositive RA, and on benralizumab for eosinophilic asthma. His RA was well controlled under this treatment.

Five days before being transferred to our institution, he presented to another hospital with high spiking fever, diarrhoea, dyspnoea/cough, and pancytopenia. Because neutropenic fever was suspected, immunosuppression was withheld, broad-spectrum antibiotics with ceftazidime were initiated, and the patient was admitted for further diagnostic workup and treatment. An episode of hypotension on the ward led to transfer to the intensive care unit, switching antibiotic therapy to piperacillin-tazobactam and fluconazole. He was successfully resuscitated with intravenous fluids, after which he was hemodynamically stable and could be transferred back to the hospitalisation ward. Abdominal ultrasound revealed splenomegaly (18.7 cm). Because of persistent fever and progressive cytopenias in combination with splenomegaly, the possibility of Felty’s syndrome complicating his RA was suggested, and he was referred to our tertiary hospital.

On admission to our hospital five days after the initial presentation, the patient remained febrile, with temperatures up to 41.0°C. Clinical examination and imaging did not identify a clear infectious focus. There were no palpable lymph nodes or signs of clinically active RA. Repeat abdominal ultrasound confirmed marked splenomegaly (Figure 1). 

**Figure 1:**
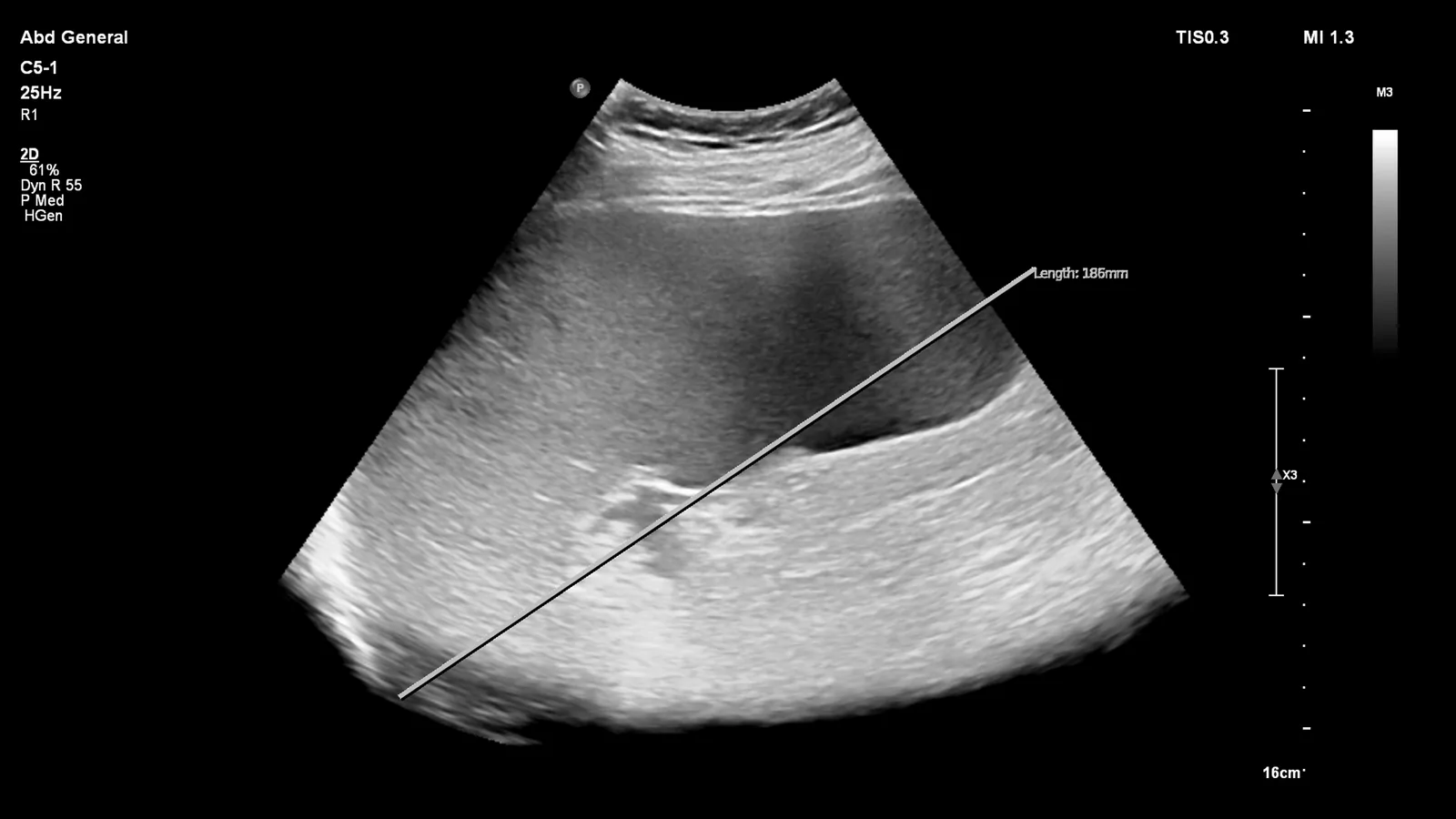
Abdominal ultrasonography in the longitudinal plane, demonstrating splenomegaly, with a maximal splenic length of 18.6 cm measured at our institution.

An extensive workup for infection and malignancy was performed. Blood, urine, and stool cultures remained negative. Pulmonary imaging showed no evidence of pneumonia. A respiratory viral panel was negative. Legionella urinary antigen testing was negative, as were HIV testing, cytomegalovirus (CMV) serology, and CMV PCR. Parvovirus B19 serology was compatible with past infection. There was no evidence of haemolysis or thrombotic microangiopathy. Peripheral blood smear and flow cytometry did not show evidence of an underlying haematologic malignancy. EBV serology was compatible with past infection, with subsequent positive EBV DNA PCR at 3.06 log IU/mL (assay linear range: 1.30-8.30 log IU/mL). As the patient had visited Spain four months earlier for two weeks, contracting several insect bites, we also tested for *Leishmania*.

Follow-up laboratory testing showed progressive pancytopenia (leucocyte nadir of 1.28 × 10⁹/L was reached at day seven), increasing inflammatory markers (peak CRP at day eight was 146.8 mg/L), transaminase, ferritin, and triglyceride concentrations, and decreasing fibrinogen, raising suspicion of secondary HLH. The overall clinical course, including the diagnostic work-up, therapeutic interventions, and evolution of ferritin and leucocyte counts, is summarised in Figure 2. Relevant laboratory results and scores are mentioned in Table 1.

**Figure 2:**
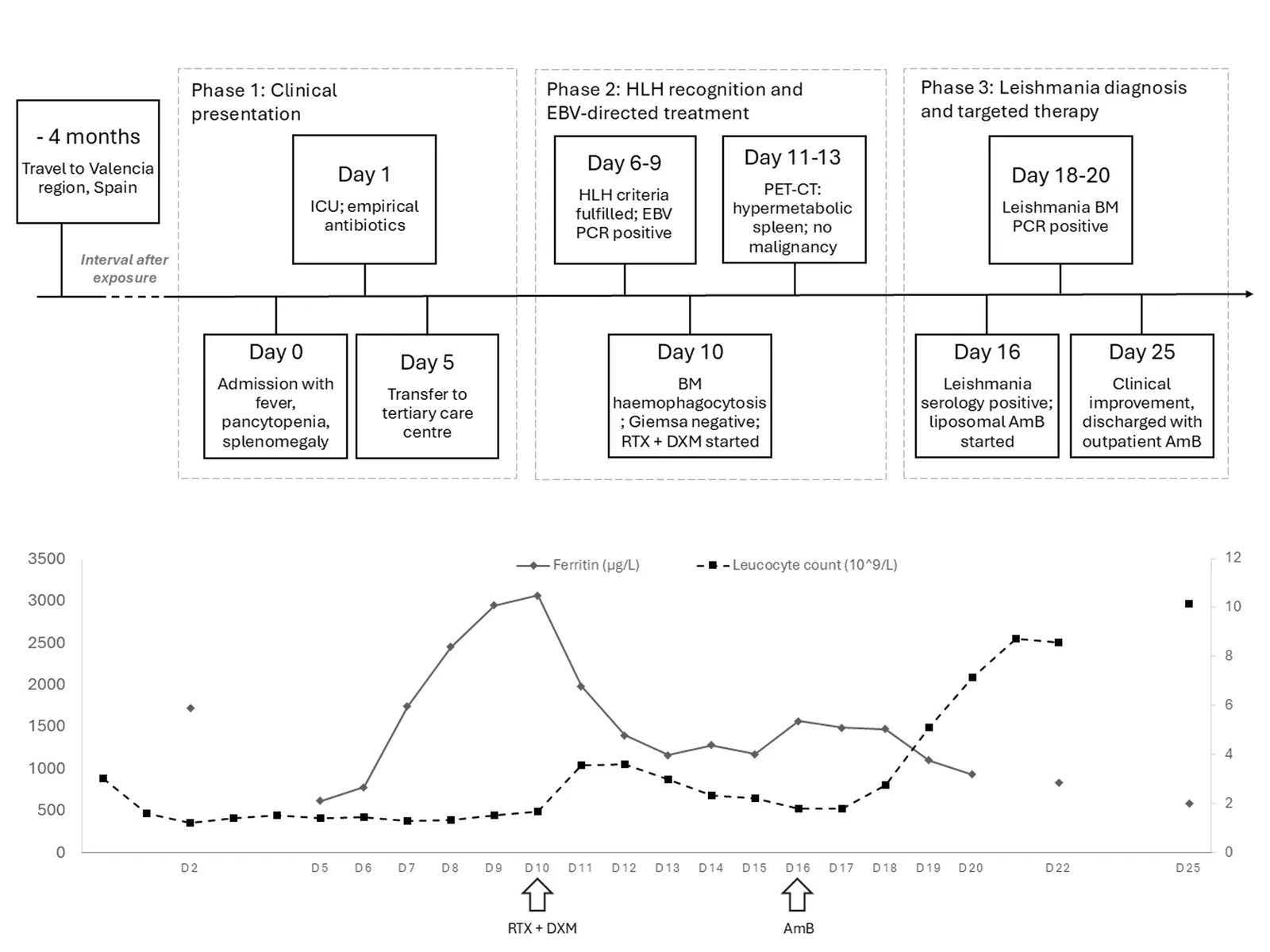
Timeline and graph of selected laboratory values Top image: Timeline of clinical presentation, diagnostic work-up, and therapeutic interventions. Lower image: Graph of selected laboratory values, showing the evolution of ferritin concentration and leucocyte count. Arrows indicate initiation of treatment with rituximab + dexamethasone and with amphotericin B. AmB, amphotericin B; BM, bone marrow; DXM, dexamethasone; EBV, Epstein-Barr virus; HLH, haemophagocytic lymphohistiocytosis; RTX, rituximab.

**Table 1:** HLH diagnostic criteria and HScore calculation in the present case *Peak or nadir values obtained during the diagnostic work-up are reported. †The sCD25 concentration was reported in pg/mL; the corresponding assay-specific threshold of ≥6,400 pg/mL was applied. ‡NK-cell activity is included in the HLH-2004 criteria but was removed from the revised 2024 diagnostic criteria. ANC, absolute neutrophil count; AST, aspartate aminotransferase; HLH, haemophagocytic lymphohistiocytosis; NA, not assessed; WBC, white blood cell.

Clinical or laboratory feature	Criterion/scoring threshold	Patient finding* (laboratory reference values)	HLH-2004 [24]	Revised HLH-2024 [25]	HScore category (points) [26]
Fever	≥38.5°C	Max 41.0°C	Met	Met	>39.4°C (49)
Splenomegaly/organomegaly	Clinical or imaging-confirmed splenomegaly	Imaging-confirmed splenomegaly, max 18.7 cm	Met	Met	Splenomegaly, no hepatomegaly (23)
Cytopenias (affecting ≥2 of 3 lineages in the peripheral blood)	Haemoglobin (<9.0 g/dL)	Haemoglobin 7.0 g/dL (14.0-18.0)	Met	Met	Three lineages (34)
	WBC ≤5.0 x 10⁹/L; ANC <1.0 x 10⁹/L	WBC 1.28 x 10⁹/L (4.00-10.00); ANC 0.697 x 10⁹/L (1.340-5.860)			
	Platelets (<100 x 10⁹/L)	Platelets 29 x 10⁹/L (150-450)			
Hypertriglyceridaemia and/or hypofibrinogenaemia	Triglycerides ≥265 mg/dL and/or fibrinogen ≤1.5 g/L	Triglycerides 309 mg/dL (≤150)	Met	Met	Triglycerides 1.5-4.0 mmol/L (44)
		Fibrinogen 0.67 g/L (2.00-3.93)			Fibrinogen ≤2.5 g/L (30)
Haemophagocytosis	Demonstrated in bone marrow or other tissue	Present in bone marrow aspirate and biopsy	Met	Met	Present (35)
Hyperferritinaemia	≥500 µg/L	3,056 µg/L (30-400)	Met	Met	2,000-6,000 µg/L (35)
Elevated soluble CD25^†^	≥2,400 U/mL or ≥6,400 pg/mL	sCD25 20,191 pg/mL	Met	Met	-
Low or absent NK-cell activity^‡^	Reduced or absent	NA	NA	-	-
Known underlying immunosuppression	-	Methotrexate and etanercept treatment	-	-	Present (18)
Elevated AST	>30 U/L	AST 176 U/L (<37)	-	-	>30 U/L (19)

Before the bone marrow examination was performed, the HScore was 252 (Table 1), providing strong support for secondary HLH and prompting the performance. Bone marrow aspiration and biopsy demonstrated haemophagocytosis (Figure 3) without evidence of an underlying haematological malignancy; no *Leishmania amastigotes* were identified on Giemsa staining. Adding haemophagocytosis to the other clinical and laboratory features increased the HScore to 287 points (corresponding to an estimated probability of HLH exceeding 99% according to the original Fardet et al. validation cohort in 2014) [26]. The patient fulfilled seven of eight HLH-2004 criteria and all seven revised HLH-2024 clinical criteria (Table 1). NK-cell activity was not assessed and is no longer included in the revised HLH-2024 criteria. The presence of secondary HLH together with detectable EBV DNA initially raised concern for EBV-associated HLH. On day 10, rituximab 700 mg (approximately 375 mg/m² BSA, the conventional dose used in EBV-associated HLH [27]; body surface area 1.91 m² by the Du Bois formula) was administered to target potentially EBV-infected B cells, while dexamethasone 10 mg/m² was initiated to suppress the hyperinflammatory HLH syndrome. The clinical and biochemical condition of the patient gradually improved in the following days, becoming afebrile.

**Figure 3:**
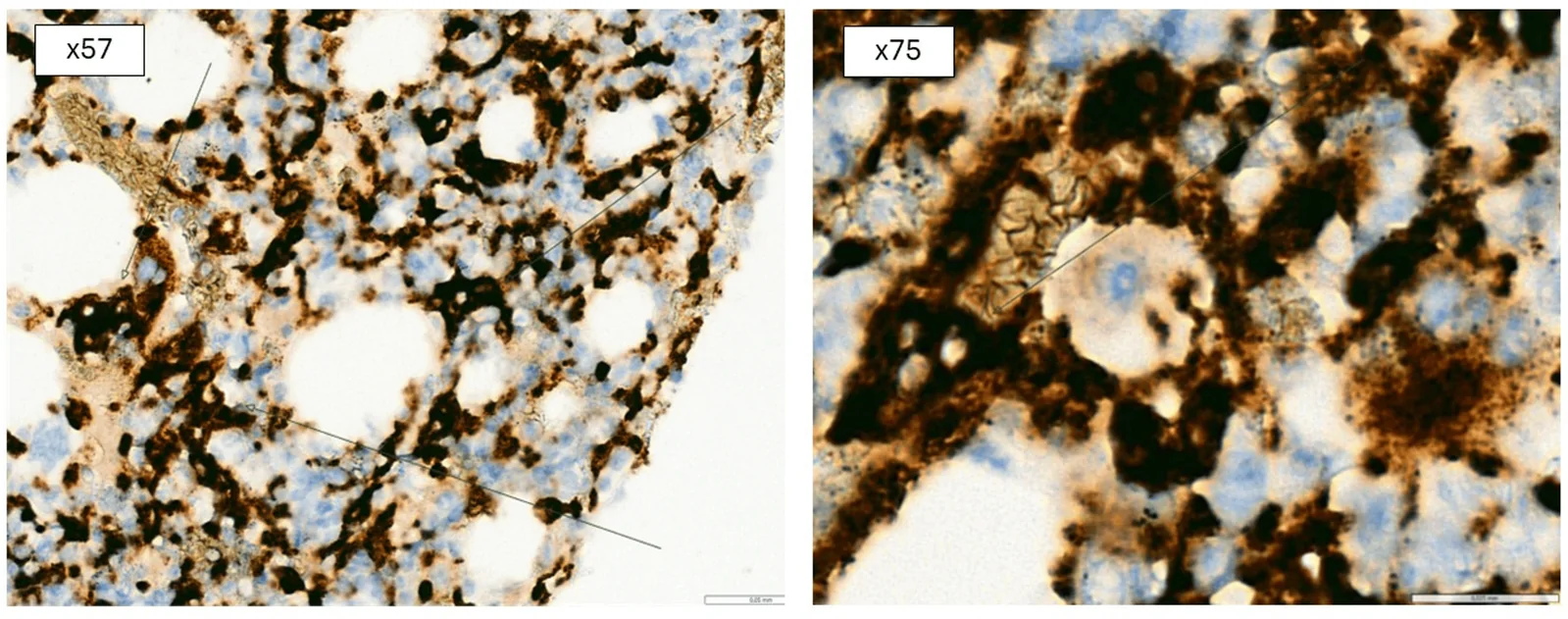
Bone marrow CD68 immunohistochemical staining demonstrating haemophagocytosis. Bone marrow sections stained for CD68, a macrophage marker, are shown at x57 magnification (left) and x75 magnification (right). Arrows indicate non-CD68-staining haematopoietic cells engulfed within CD68-positive macrophage cytoplasm, consistent with haemophagocytosis.

Six days later, *Leishmania* serology results returned strongly positive. Given the compatible clinical syndrome and recent epidemiological exposure, VL was suspected. *Leishmania* PCR was subsequently performed on the same bone marrow aspirate that had previously been examined. Repeat EBV PCR showed that the viral load had lowered (1.85 log IU/mL). 

Liposomal amphotericin B at 4 mg/kg intravenously was started, on days 1-5, 10, 17, 24, 31, and 38, for a total cumulative dose of 40 mg/kg. Liposomal amphotericin B is the preferred treatment for VL in this immunocompromised patient; alternative antileishmanial agents include miltefosine and sodium stibogluconate, but these are considered when first-line treatment is not available or tolerated [7].

*Leishmania* PCR on bone marrow returned positive for *L. infantum*, confirming the diagnosis of VL, despite the absence of visible amastigotes on Giemsa-stained microscopy. The corticosteroids were tapered. After starting antileishmanial treatment, the patient remained afebrile and showed decreasing CRP and ferritin levels and progressive recuperation of leucocyte and platelet counts. The patient was discharged after day 10 of liposomal amphotericin B, and treatment was continued via the day hospital. 

At follow-up after completing the therapy, blood counts and inflammatory markers remained normal, and ultrasonography confirmed normalisation of spleen size. At two months evaluation, his RA remained in remission (DAS28-CRP 2.3). Methotrexate was restarted, whereas etanercept was not resumed. 

## Discussion

Methods

On 16 May 2026, one reviewer (FF) performed a targeted narrative literature search in PubMed/MEDLINE from database inception to the search date. The review aimed to identify published adult cases of (1) VL in patients with RA, (2) VL-associated HLH in immunosuppressed patients with immune-mediated inflammatory diseases, and (3) VL or VL-HLH cases in which suspicion of EBV infection/reactivation confounded the diagnostic workup. This was a focused narrative review and was not conducted according to the Preferred Reporting Items for Systematic reviews and Meta-Analyses (PRISMA) methodology.

Search terms included combinations of “visceral leishmaniasis”, “Leishmania”, “Leishmania infantum”, “kala-azar”, “hemophagocytic lymphohistiocytosis”, “HLH”, “hemophagocytic syndrome”, “macrophage activation syndrome”, “rheumatoid arthritis”, “arthritis, rheumatoid”, “autoimmune diseases”, “autoimmune disease”, “rheumatic diseases”, “spondylarthritis”, “psoriasis”, “inflammatory bowel diseases”, “immune-mediated inflammatory disease”, “IMID”, “methotrexate”, “anti TNF”, “Tumor Necrosis Factor Inhibitors”, “Tumor Necrosis Factor Alpha Inhibitor”, “antirheumatic drugs”, “etanercept”, “biologic therapy”, and “Epstein-Barr virus”.

The reference lists of retrieved articles were manually screened to identify additional reports. Paediatric cases, cases limited to cutaneous/mucocutaneous leishmaniasis without visceral involvement, and non-English publications of which the abstracts did not provide sufficient information were excluded.

Results and discussion

We identified 23 reported adult cases of VL in patients with RA [14-17,19,20,28-44], three cases of VL-HLH in immunosuppressed patients with immune-mediated inflammatory diseases [5,13,17], and four patients in whom EBV findings complicated the diagnostic interpretation, but VL was ultimately identified as the principal trigger [45-48]. The features of the cases reporting on VL-HLH in immunosuppressed patients and cases with EBV as a potential confounder are summarised in Table 2. Across reports, fever, splenomegaly, pancytopenia, hyperferritinaemia, and hepatitis were common presenting features. Several cases also demonstrated that initial bone marrow evaluation may be negative or misleading, and that repeated tissue evaluation, serology, and PCR may be required to establish the diagnosis of leishmaniasis.

**Table 2:** Current and previously published adult cases of visceral leishmaniasis-associated HLH with immunosuppression or EBV-related diagnostic confounding AmB, amphotericin B; BM, bone marrow; EBV, Epstein-Barr virus; HBV/HCV, hepatitis B/C virus; HLH, haemophagocytic lymphohistiocytosis; IVIG, intravenous immunoglobulin; LN: lymph node, mNGS, metagenomic next-generation sequencing; MTX, methotrexate; NR, not reported (this marks details not reported in the cited paper); PCR, polymerase chain reaction; RA, rheumatoid arthritis; TNF, tumour necrosis factor; VL, visceral leishmaniasis; VP-16, etoposide.

Author, year	Country/exposure	Age/sex	Underlying disease	Immunosuppression	EBV or viral finding	*Leishmania* diagnostic method	Microscopy positive	HLH treatment	Anti-leishmanial treatment	Outcome	Main lesson	
Current case report												
Fleurinck et al., 2026	Belgium/Valencia, Spain, four months earlier; insect bites	59/M	Seropositive RA; eosinophilic asthma	MTX, etanercept, benralizumab	Low-titre EBV PCR; initially suspected EBV-HLH	Serology positive; bone marrow PCR positive	Giemsa stain did not reveal amastigotes.	Rituximab + dexamethasone initially	Liposomal amphotericin B	Favourable early response	EBV reactivation may misdirect diagnosis and may be secondary; leishmaniasis PCR crucial	
VL-HLH in immunocompromised patients												
Moltó et al., 2010 [17]	Spain	60/M	Rheumatoid arthritis	Adalimumab	No specific EBV driver reported	Intra-macrophagic *Leishmania* amastigotes, no haemophagocytosis seen	Yes	None; HLH suspected (fulfilling HLH-2004 criteria), no haemophagocytosis on BM	Liposomal amphotericin B	Clinical and biochemical recovery, no amastigotes at repeat BM	Absence of haemophagocytosis on BM exam does not exclude HLH	
Bukan et al., 2019 [5]	Denmark/no recent endemic travel; dormant VL proposed (LN excised one year prior showed amastigotes)	68/M	Sarcoidosis	Infliximab, prednisolone, and methotrexate	Low-titre EBV viral load; argued to be secondary phenomenon	Serology borderline positive; bone marrow: haemophagocytosis and L. amastigotes + bone marrow PCR positive	Yes	Dexamethasone, intravenous immunoglobulins. No etoposide. No rituximab (initially considered for EBV-HLH)	No amphotericin B	Death, VL considered primary trigger	In HLH after anti-TNF therapy, test for VL; EBV viraemia may be secondary (co-infection) or contributed to immune dysregulation and the hyperinflammatory state.	
Estabrooks et al., 2023 [13]	Australia/brief trip to India >20 years earlier	56/M	Ulcerative colitis	Infliximab	No specific EBV driver reported	Initial BM: haemophagocytosis only; later colonic biopsy consistent with *Leishmania* and PCR confirmed; repeat BM with Leishman-Donovan bodies	Initially no; later yes	Etoposide + dexamethasone + cyclosporin initially	Liposomal amphotericin B	Full recovery after prolonged admission	Remote travel can matter; continue diagnostic surveillance if HLH trigger is not identified.	
Initially suspected EBV-HLH or EBV-related diagnostic confounding												
Gaifer et al., 2016 [45]	Oman/travel to Yemen seven months earlier	27/M	Previously healthy	None	High EBV viraemia (13.55 x 10³ IU/mL) and positive EBV serology	Positive L. infantum serology; BM aspirate/biopsy showed Leishman-Donovan bodies and haemophagocytosis	Yes	None; treated causally	Liposomal amphotericin B	Complete resolution of symptoms and clearance of EBV viraemia	Treating VL alone may resolve HLH and EBV viraemia; EBV may not be the principal driver.	
Neycheva et al., 2021 [46]	Bulgaria/exposure NR (endemic in Bulgaria)	40/F	Previously healthy	None before illness	Chronic EBV infection initially interpreted as trigger (IgG positive; IgM grey zone; PCR negative)	Amastigotes were present but missed on first BM; repeat BM later showed numerous *Leishmania* parasites	Yes, but initially unrecognised	Cyclosporin A + corticosteroids; IVIG also used	Antileishmanial therapy after diagnosis (meglumine antimoniate)	Temporary response, then relapse; diagnosis revised to *Leishmania*-triggered HLH	Careful marrow review is critical; missed amastigotes can lead to unnecessary immunosuppression.	
Potapenko et al., 2022 [47]	Russia/travel to Spain 1.5 months earlier	30/F	NR	None reported before illness	Positive EBV capsid IgG; treated as presumed EBV-HLH	Splenic leishmaniasis found only after diagnostic splenectomy.	Yes in spleen	HLH-2004 chemotherapy for presumed EBV-HLH	Amphotericin B	Cured after VL diagnosis and amphotericin B	Travel-related VL can masquerade as EBV-HLH; splenic/BM tissue diagnosis may be decisive.	
Wang et al., 2025 [48]	China/travel to Xinjiang (China) six months earlier	31/M	Previously healthy	None	EBV-DNA 9.79 x 10^3^ IU/mL after initial negative EBV; acyclovir ineffective	Peripheral-blood mNGS positive for *Leishmania* spp.; BM showed haemophagocytosis but no amastigotes.	No amastigotes on BM smear	Dexamethasone + etoposide (VP-16)	Amphotericin B deoxycholate	Recovered; no recurrence during two-year follow-up	EBV positivity may coexist with VL-HLH; mNGS can identify VL when microscopy is negative.	

HLH as a Syndrome, Not a Final Diagnosis

Diagnosing HLH is not an end point: urgent aetiological clarification is required because treatment depends on the underlying cause or trigger. In adults, HLH may be associated with severe infection, malignancy and autoimmune disease, while these diseases can produce overlapping clinical and laboratory abnormalities [1]. In patients with immune-mediated inflammatory diseases, disease-specific complications may further complicate the differential diagnosis. In RA, for example, fever, splenomegaly, and cytopenias may initially suggest Felty’s syndrome, a rare complication of longstanding RA characterised by the triad of RA, neutropenia, and splenomegaly [49].

In clinical practice, several diagnostic frameworks are used for recognising secondary HLH. The HScore provides a continuous estimate of the probability of adult secondary HLH, with ≥169 commonly used as a supportive diagnostic threshold, although higher scores -- especially ≥200 -- provide stronger evidence, and clinical context remains essential [26,50,51]. The HLH-2004 criteria were originally developed for the diagnosis of familial and paediatric HLH but are now widely used to support the diagnosis of HLH in clinical practice [24]. The revised HLH-2024 criteria were introduced to update and simplify this diagnostic framework [25]. Neither framework identifies the underlying cause of HLH.

VL and HLH share several clinical and biological features, making VL-associated HLH difficult to distinguish from severe VL. Conversely, once HLH has been recognised, confirmation of VL does not automatically establish that *Leishmania* is the sole trigger, particularly in immunosuppressed patients in whom alternative or concomitant triggers may be present [52]. Ferritin is likewise nonspecific, and infection-associated HLH should not be dismissed solely because ferritin concentrations are only moderately elevated when the overall clinical and laboratory pattern is convincing.

In the present case, the diagnosis of VL-associated HLH was supported by an HScore of 287 points, fulfilment of seven of eight HLH-2004 criteria, and all seven revised HLH-2024 clinical criteria. Subsequent positive *Leishmania* serology and detection of *Leishmania* DNA in bone marrow established concomitant VL. The clinical course, fulfilment of established HLH criteria, and microbiological confirmation of VL were consistent with VL-associated HLH. Detectable EBV DNA nevertheless raised the possibility of an additional trigger.

Identifying the infectious trigger has direct therapeutic implications. Published cases suggest that timely antileishmanial treatment alone can reverse HLH in some patients, whereas critically ill patients may require additional immunomodulatory therapy [53]. Early treatment may therefore avoid escalation to more toxic HLH-directed regimens [22,47]. In our patient, dexamethasone and rituximab may have contributed to the initial improvement. However, sustained recovery after initiation of antileishmanial therapy favoured VL as the principal HLH trigger.

EBV Reactivation as a Diagnostic Pitfall

Following primary infection, EBV persists latently and may intermittently replicate, including at low levels in otherwise asymptomatic individuals. In immunosuppressed patients or during severe systemic inflammation, the detection of EBV DNA in peripheral blood may therefore reflect reactivation or bystander replication, rather than necessarily representing the primary HLH trigger. The clinical significance of EBV PCR positivity depends on the magnitude and kinetics of the viral load, interpreted against assay-specific thresholds and within the overall clinical context [54].

In our patient, EBV DNAemia initially supported EBV-associated HLH as a working diagnosis and prompted treatment with rituximab and dexamethasone. However, in retrospect, the relatively low viral load, together with eventual confirmation of *Leishmania* infection and clinical response to anti-leishmanial therapy, supported VL as the more plausible principal trigger. Treating presumed EBV-HLH, although being clinically defensible, could have delayed VL diagnosis.

As summarised in Table 2, similar diagnostic confounding has been reported in other cases, including patients in whom EBV DNA became undetectable following treatment of VL, even without specific EBV-directed therapy [22,47], suggesting that its detection may reflect secondary immune dysregulation. However, EBV may still contribute in individual cases. EBV positivity, even at higher viral loads, should therefore not end the diagnostic work-up when clinical features, travel history, or immunosuppression suggest an alternative infectious trigger.

Diagnosing VL: Molecular Diagnostics to the Rescue

In this patient, bone marrow microscopy alone was insufficient to diagnose VL. Bone marrow examination demonstrated haemophagocytosis, but Giemsa staining did not show *L. amastigotes*. The diagnosis of VL was only established after *Leishmania* serology became positive, followed by bone marrow PCR.

Several published cases similarly show that initial bone marrow evaluation may be non-diagnostic or misleading, possibly due to low parasite burden or patchy involvement [13,55]. Although negative bone marrow microscopy does not exclude VL, bone marrow aspirate and biopsy remain essential in patients presenting with fever, cytopenias, and splenomegaly. They allow exclusion of underlying haematologic disease and may demonstrate haemophagocytosis while also providing material for molecular testing when VL remains clinically suspected. In some cases, repeated bone marrow examination, alternative tissue sampling, serology, and* Leishmania* PCR on blood, bone marrow, and other tissues may be required.

The IDSA/ASTMH guidelines recommend combining multiple diagnostic modalities in suspected leishmaniasis, including microscopy, histopathology, culture, molecular detection of parasite DNA and, for VL, serology [6]. These guidelines also state that molecular amplification assays are among the most sensitive tests; they also recommend bone marrow aspiration as the preferred first diagnostic sample in suspected VL [6]. Furthermore, serology remains positive for a long time; therefore, it alone is not evidence of ongoing infection and should trigger further testing [56].

VL, VL-HLH, and Immunosuppression in Patients With Rheumatologic Diseases

In our patient, several factors may have increased susceptibility to *Leishmania* infection: RA on methotrexate, TNF inhibition with etanercept, and recent travel to the Mediterranean region. Benralizumab was also part of our patient’s immunomodulatory background treatment, although a causal association between anti-IL-5 therapy and VL has never been established.

VL has repeatedly been reported in both immunocompetent and immunosuppressed individuals, either as a primary infection (after recent exposure) or as reactivation of latent disease (in some reports several decades earlier) [5,11-21,43,57]. Table 2 summarises reported adult cases in which VL-HLH occurred in the setting of immunosuppression and rheumatic disease. Together, these reports suggest that *Leishmania *should be considered in immunosuppressed patients presenting with fever, pancytopenia, and splenomegaly, particularly after compatible exposure.

The role of TNF inhibition in increasing the risk of VL is biologically plausible, as TNF is part of the cell-mediated defence, normally containing *Leishmania* through granuloma formation, macrophage activation, and Th1-type immunity [12,58-60]. Blocking TNF makes latent or newly acquired *Leishmania* infection harder to control.

Reliable estimates of the risk of VL specifically in rheumatological patients receiving TNF inhibitors are lacking, as the available evidence consists largely of case reports and small series. More quantitative data are available in inflammatory bowel disease. A recent Spanish study found a 13.2-fold higher incidence of symptomatic leishmaniasis in IBD patients compared with the general population (however, predominantly cutaneous disease was observed), with anti-TNF therapy being the principal driver of infection risk [61]. The monoclonal antibodies infliximab and adalimumab seem to confer a higher risk than etanercept (drug-specific relative risk for opportunistic leishmaniasis: 8-fold increased odds (p=0.0096) [62]); this might be due to their stronger effects on transmembrane TNF [58,62].

Given the widespread use of anti‐TNF and the current state of international tourism, physicians prescribing anti‐TNF therapy should be aware of the risk of leishmaniasis, regardless of their geographical location. Whenever a patient presents with suggestive symptoms, they should consider potential local exposure and, in non-endemic regions, both recent and distant travel history.

Limitations

This case report has several limitations. First, it describes a single case and cannot establish a causal relationship between the development of VL and individual immunosuppressive therapies. Although VL was considered the principal trigger of HLH, a contributory role of EBV cannot be excluded. Second, the accompanying literature review was focused and narrative rather than systematic, was performed by a single reviewer, and was not conducted according to the PRISMA methodology; publication and selection bias are therefore possible. Third, NK-cell activity was not assessed, although this parameter is no longer included in the revised HLH-2024 criteria. Fourth, because of the early improvement after rituximab and dexamethasone administration, the subsequent clinical course cannot be attributed exclusively to antileishmanial treatment. Finally, follow-up was limited to two months, and no post-treatment *Leishmania* PCR on bone marrow was performed; microbiological eradication was therefore not documented. These limitations preclude broader causal or therapeutic conclusions from this individual case.

## Conclusions

VL should be considered an important and treatable trigger of HLH in immunosuppressed patients with compatible recent or remote epidemiological exposure, be it habitation or travel. Detectable EBV DNA may reflect bystander replication or reactivation but should not automatically be considered the principal cause of HLH and should not terminate the search for alternative infectious causes. When VL remains clinically plausible, negative microscopy should prompt complementary diagnostic testing, including serology and molecular detection of *Leishmania* DNA.

The learning points include (1) HLH is a hyperinflammatory syndrome; its diagnosis does not establish the underlying trigger. (2) Detectable EBV DNA may represent reactivation or a coexisting process rather than the principal cause of HLH. (3) Negative bone marrow microscopy does not exclude VL; serology and molecular testing may be required. (4) Both recent and remote exposure to *Leishmania*-endemic regions, including Mediterranean Europe, should be considered in immunosuppressed patients.
